# General Anesthesia and Systemic Hyperosmolality Modulate Lumbar Intrathecal Drug Distribution in Female Rats

**DOI:** 10.1097/ALN.0000000000005794

**Published:** 2025-10-09

**Authors:** Niklas Daniel Åke Persson, Terhi J. Lohela, Jenni E. Anttila, Jenni Mäkelä, Marko Rosenholm, Martta Peltoniemi, Sonja C. Jalonen, J. Arturo García-Horsman, Mirkka Sarparanta, Maiken Nedergaard, Tomi Rantamäki, Tuomas O. Lilius

**Affiliations:** 1 Individualized Drug Therapy Research Program and Department of Pharmacology, Faculty of Medicine, and Division of Pharmacology and Pharmacotherapy, Drug Research Program, Faculty of Pharmacy, University of Helsinki, Helsinki, Finland.; 2 Individualized Drug Therapy Research Program, Faculty of Medicine, and Division of Pharmacology and Pharmacotherapy, Drug Research Program, Faculty of Pharmacy, University of Helsinki, Helsinki, Finland; Department of Anaesthesiology, Intensive Care and Pain Medicine, HUS Helsinki University Hospital and University of Helsinki, Helsinki, Finland.; 3 Individualized Drug Therapy Research Program, Faculty of Medicine, and Division of Pharmacology and Pharmacotherapy, Drug Research Program, Faculty of Pharmacy, University of Helsinki, Helsinki, Finland.; 4 Individualized Drug Therapy Research Program, and Department of Pharmacology, Faculty of Medicine, University of Helsinki, Helsinki, Finland.; 5 Division of Pharmacology and Pharmacotherapy, Drug Research Program, Faculty of Pharmacy, University of Helsinki, Helsinki, Finland.; 6 Individualized Drug Therapy Research Program, Faculty of Medicine, and Division of Pharmacology and Pharmacotherapy, Drug Research Program, Faculty of Pharmacy, University of Helsinki, Helsinki, Finland.; 7 Individualized Drug Therapy Research Program, Faculty of Medicine, and Division of Pharmacology and Pharmacotherapy, Drug Research Program, Faculty of Pharmacy, University of Helsinki, Helsinki, Finland.; 8 Division of Pharmacology and Pharmacotherapy, Drug Research Program, Faculty of Pharmacy, University of Helsinki, Helsinki, Finland.; 9 Department of Chemistry, Radiochemistry, University of Helsinki, Helsinki, Finland.; 10 Center for Translational Neuromedicine, Faculty of Health and Medical Sciences, University of Copenhagen, Copenhagen, Denmark; and Center for Translational Neuromedicine, University of Rochester Medical Center, Rochester, New York.; 11 Division of Pharmacology and Pharmacotherapy, Drug Research Program, Faculty of Pharmacy, and SleepWell Research Program, Faculty of Medicine, University of Helsinki, Helsinki, Finland.; 12 Individualized Drug Therapy Research Program, and Department of Pharmacology, Faculty of Medicine, and Division of Pharmacology and Pharmacotherapy, Drug Research Program, Faculty of Pharmacy, University of Helsinki, Helsinki, Finland; Department of Emergency Medicine and Services, HUS Helsinki University Hospital and University of Helsinki, Helsinki, Finland.

## Abstract

**Background:**

Spinal administration of drugs largely bypasses the blood–brain barrier that reduces the central nervous system (CNS) availability of most systemically administered drugs. Current methods for lumbar intrathecal drug delivery fail to deliver therapeutic concentrations to the brain, whereas intracranial drug administration carries marked risks. Cerebrospinal fluid flow along the glymphatic pathway can be pharmacologically modified with a variety of drugs including anesthetics and hypertonic saline (HTS). However, the effect of anesthetics and HTS on spinally administered drugs is highly understudied. The authors investigated how two anesthetic regimens and HTS influence the distribution of a spinally administered high-molecular-weight radiotracer.

**Methods:**

Female rats were anesthetized with ketamine–dexmedetomidine or isoflurane. HTS (40 mOsm/kg) or isotonic saline (ITS) was administered intraperitoneally. The whole-body distribution of lumbar spinal tracer (technetium-99m radiolabeled human serum albumin nanocolloid, 66.5 kDa) was assessed with *in vivo* single-photon emission computed tomography. Anesthetic-induced changes in spinal subarachnoid space volume were investigated with magnetic resonance imaging.

**Results:**

Compared with isoflurane, ketamine–dexmedetomidine enhanced local spinal tracer availability (area under the time–activity curve between 0 and 116 min [AUC_0-116_] ratio, 1.78; *P* = 0.0016) and reduced intracranial exposure (AUC_0-116_ ratio, ∞; *P* = 0.0260) in ITS-treated rats. Moreover, ketamine–dexmedetomidine increased the spinal subarachnoid space volume (T13–T6) by 46% (*P* = 0.0051) compared with isoflurane. HTS markedly increased intracranial tracer availability compared with ITS during ketamine–dexmedetomidine anesthesia (AUC_0-116_ ratio, ∞; *P* = 0.0047) but not isoflurane (AUC_0-116_ ratio, 3.10; *P* = 0.1320), and prolonged CNS retention in awake rats.

**Conclusions:**

Anesthesia modulates the distribution of intrathecally administered drugs at the spinal and intracranial levels. Systemic HTS increased the intracranial availability of spinally administered drugs during ketamine–dexmedetomidine and prolonged the CNS availability of spinal drugs in the awake state. These interventions should be taken to clinical trials to improve efficacy and to reduce side effects of spinally administered drugs.

## Abstract 2

In female rats, magnetic resonance imaging revealed that ketamine–dexmedetomidine anesthesia increased spinal subarachnoid space volume when compared to isoflurane anesthesia. Single–photon emission computed tomography revealed that the increase in spinal subarachnoid space under ketamine–dexmedetomidine anesthesia was correlated with a restricted distribution of a radioactive tracer away from the infusion site while isoflurane promoted tracer distribution along the spinal canal toward the intracranial space. Systemic administration of hypertonic saline increased radioactive tracer distribution toward the intracranial space under ketamine–dexmedetomidine but not under isoflurane anesthesia.

## Editor’s Perspective

**What We Already Know about This Topic**

- Preclinical studies suggest that increasing serum osmolality *via* hypertonic saline administration could enhance brain delivery of intrathecally administered drugs
- There is limited information of how general anesthetics influence the intracranial distribution of drugs injected into the cerebrospinal fluid space at the lumbar level, and it remains unknown whether systemic hypertonic saline administration can influence this process

**What This Article Tells Us That Is New**

- In female rats, magnetic resonance imaging revealed that ketamine–dexmedetomidine anesthesia increased spinal subarachnoid space volume when compared to isoflurane anesthesia
- Single-photon emission computed tomography revealed that the increase in spinal subarachnoid space under ketamine–dexmedetomidine anesthesia was correlated with a restricted distribution of a radioactive tracer away from the infusion site while isoflurane promoted tracer distribution along the spinal canal toward the intracranial space.
- Systemic administration of hypertonic saline increased radioactive tracer distribution toward the intracranial space under ketamine–dexmedetomidine but not under isoflurane anesthesia.

A variety of drugs can be administered into the spinal cerebrospinal fluid (CSF) space to largely bypass the blood–brain barrier that dramatically reduces the central nervous system (CNS) availability of systemically administered drugs,^1^ including gene therapy,^2,3^ antinociceptive drugs and spinal anesthetics,^4^ antimicrobials,^5^ drugs for spasticity,^6^ and cancer chemotherapy.^7^ Further, the significance of direct CSF administration is likely to increase in the future due to an increasing number of CNS therapeutics with large molecular size and progress in cisterna magna (CM) administration techniques.^2,3^ While the distribution of CM-administered drugs can be pharmacologically modified, ways to modify the distribution of spinally administered drugs are largely unexplored.

Compared with direct administration into the CM or the lateral ventricles, the lumbar route is associated with fewer complications and requires no neurosurgical expertise.^8,9^ However, only few studies have investigated how effectively spinally administered drugs reach the brain. While some studies have showed relatively high delivery of small molecular tracers in humans,^10,11^ clinical trials with large molecular drugs have failed to reach therapeutic concentrations in the brain, hindering CNS drug development.^12^ Further, preclinical studies have found limited cerebral availability in rats anesthetized with isoflurane (ISO) or pentobarbital.^13,14^ Hence, enhancing the cranial distribution of spinal drugs could be an attractive approach for treating pathologies of the upper spinal canal or the brain.

Recent discoveries on the glymphatic system have opened new opportunities for drug delivery to the CNS. The glymphatic pathway refers to directional CSF flow along the periarterial Virchow–Robin spaces into the CNS parenchyma and concurrent perivenous efflux to systemic circulation. The glymphatic influx promotes CNS drug delivery, while efflux supports clearance.^15^ While the glymphatic influx has been found to be low under ISO, α_2_-adrenergic agonists (dexmedetomidine or xylazine), in combination with either ketamine or ISO^16–20^ and an increase in plasma osmolality, *e.g.*, with hypertonic saline (HTS) or hyperosmolar mannitol,^21–25^ have been shown to promote CSF influx and drug delivery into the brain after CM or intraventricular administration. However, the effect of neither anesthesia nor HTS on CSF dynamics and drug delivery has been assessed after lumbar intrathecal administration.

We have recently introduced the use of single-photon emission computed tomography (SPECT), a nuclear medicine tomographic imaging technique using gamma rays, for quantitative whole-body assessment of CSF tracer distribution and pharmacokinetics.^16^ We investigated here how (1) the choice of anesthesia (ketamine–dexmedetomidine [K/DEX] *vs.* ISO) and (2) HTS compared with isotonic saline (ITS) modify the distribution of a spinally administered isobaric macromolecular radiotracer, serving as a surrogate for macromolecular drugs such as antibodies and protein-based drugs (technetium-99m radiolabeled human serum albumin nanocolloid, [^99m^Tc]Tc-NanoHSA; molecular weight, 66.5 kDa) by performing dynamic SPECT in adult female rats. Further, we used magnetic resonance imaging (MRI) to investigate how the volume of the compartments of the spinal canal are affected by the anesthetic regimens. Based on previous studies on CSF distribution during different anesthetic regimens^16,17^ and systemic HTS^21,22^ after CM infusion, we hypothesized that the cranial distribution of lumbar spinal drugs would be enhanced by systemic HTS and that anesthetic choice would influence the tracer distribution.

## Materials and Methods

### Animals

Experiments were approved by the Southern Finland Regional State Administrative Agency (Hämeenlinna, Finland; No. ESAVI/36258/2020). Female Sprague–Dawley rats (n = 49; mean ± SD, 223 ± 26 g; Envigo, The Netherlands; or Janvier Laboratories, France) were group-housed four per cage with *ad libitum* access to food and water in a temperature-controlled environment (23° ± 2°C) with a 12-h/12-h light/dark cycle. Rats receiving a chronic cannula were housed individually. The experiments were conducted during the light phase. The estrous cycle was not measured as we presumed that any effect by the estrous cycle would be overcome by the interventions. This assumption is supported by the lack of difference between the sexes in glymphatic CSF flow.^26^

### Lumbar Catheterization

The rats were anesthetized with either a mixture of K/DEX containing ketamine (100 mg/kg; Ketaminol vet 50 mg/ml; Intervet, The Netherlands or Ketaminol vet 100 mg/ml; Intervet, The Netherlands) and dexmedetomidine (0.5 mg/kg; Dexdomitor 0.5 mg/ml; Orion Pharma, Finland) by subcutaneous injection (2 ml/kg) or by ISO (3% induction; 1.5 to 2% maintenance; Vetflurane, Virbac, France), similar to previous studies.^16,17,21,22^ After a loss of toe-pinch reflex, a lumbar catheter was placed as previously described (Supplemental Digital Content Methods 1, https://links.lww.com/ALN/E274).^27^ For dynamic SPECT ,the protocol was modified such that the catheter was cut with only 8 cm dead volume and directly exposed at the back of the rat.

### SPECT Imaging

Detailed information for the radiotracer production and SPECT imaging is included in Supplemental Digital Content Methods 1 (https://links.lww.com/ALN/E274). [^99m^Tc]Tc-nanoHSA (ROTOP Pharmaka, Germany; molecular weight, 66.5 kDa) was used as a lumbar intrathecal CSF tracer (infusion volume of 25 μl at an infusion rate of 2 μl/min). For dynamic SPECT, a computed tomography (CT) reference image was collected with nine consecutive dynamic whole-body SPECT acquisitions more than 116 min. Infusion of [^99m^Tc]Tc-nanoHSA started at the beginning of second frame. HTS (1 M NaCl; 20 ml/kg equivalent to 40 mOsm/kg) or ITS (0.154 M NaCl; control group) was injected intraperitoneally more than 2 min directly after CSF tracer infusion. The dose of HTS was based on previous studies to allow a comparison of results with previous data.^21,22,24^ Groups were studied with dynamic SPECT K/DEX-ITS (n = 6), K/DEX-HTS (n = 7), ISO-ITS (n = 9), and ISO-HTS (n = 7). The sample size was estimated based on previous studies on the influence of anesthetics or hypertonic solutions on CSF distribution imaged with SPECT in rats.^16,17,21,22^ While the order of the anesthetic was not randomized, the order of the hypertonic intervention was random, with both ITS- and HTS-treated animals imaged during each experimental day. Experimental data were excluded from further analysis due to failed tracer infusions (n = 6) or failed image acquisition (n = 1). No further animals or data points were excluded during the analysis. For 24-h SPECT acquisitions, rats with a chronic catheter were anesthetized with ISO, and sutures over the L4–L6 were removed to expose the catheter. HTS or ITS was injected intraperitoneally, and a CT reference image was collected with one consecutive full-body SPECT acquisition. Infusion of [^99m^Tc]Tc-NanoHSA started concurrently with the first CT acquisition. CT/SPECT acquisition was repeated during brief ISO anesthesia 3, 6, and 24 h after tracer infusion, and the rat was awake in the home cage between scans. The following groups were studied for 24-h ITS: (n = 6) and HTS (n = 6). The sample size was estimated based on previous studies on the influence of anesthetics or hypertonic solutions on CSF distribution imaged with SPECT in rats.^16,17,21,22^ The order of the hypertonic intervention was random with both ITS- and HTS-treated animals imaged during each experimental day. Experimental data were excluded from further analysis due to failed tracer infusions (n = 2). No further animals or data points were excluded during the analysis.

### Magnetic Resonance Imaging

T2-weighted structural imaging of rats during K/DEX (n = 4) or ISO (n = 4) anesthesia was acquired using three-dimensional constructive interference steady state. The order of the anesthetic was randomized. Detailed information is available in Supplemental Digital Content Methods 1 (https://links.lww.com/ALN/E274).

### SPECT Data Analysis

Acquired images were reconstructed with Nucline acquisition software (Mediso, Hungary). Regions of interest (ROIs) were determined utilizing ITK-SNAP (Supplemental Digital Content Methods 1, https://links.lww.com/ALN/E274).^28^ The percentage of injected dose (%ID) in each ROI was calculated utilizing MATLAB R2022a (MathWorks, USA). Data acquisitions were performed unblinded, while ROIs were drawn blinded.

### MRI Data Analysis

All MRI data were preprocessed to calculate a three-dimensional constructive interference steady state image for each rat (Supplemental Digital Content Methods 1, https://links.lww.com/ALN/E274). ROIs were drawn over each spinal column and corresponding subarachnoid space ranging from T13–T6 utilizing the ITK-SNAP software. The percentage of the subarachnoid space volume of the spinal canal was calculated for each column individually defined as the $\frac{subarachnoid\;space\;volume}{total\;column\;volume}\times 100\%$. Data acquisitions and analyses were performed unblinded.

### Statistical Analysis

Statistical analysis and graphical representation were performed with GraphPad Prism 10 (GraphPad Software, USA). We assessed the pharmacokinetic parameters for [^99m^Tc]Tc-NanoHSA by first calculating the maximum percentage of injected dose (C_max_), the time to maximum percentage of injected dose, and the area under the time–activity curve between 0 and 116 min (AUC_0-116_) and between 0 and 24 h for each ROI in each individual animal, as well as time to first activity detected (arrival time) for ROIs other than the infusion site and directly adjacent ROIs. In addition, we calculated the ratio between ROIs located cranially and caudally (cranial-to-caudal ratio) from the infusion site within the CNS to estimate the relative direction of flow. The quantification of the spinal MRI data was done by calculating the spinal canal volume, the corresponding subarachnoid space volume, and the percentage subarachnoid space for the total field of view (T13–T6). All data are presented with median and range (minimum to maximum). Histograms and normality tests (Shapiro–Wilk test) were used to check for normality. Since most of the SPECT data comparisons were not normally distributed, all comparisons were made using two-tailed Mann–Whitney tests. The structural MRI data were normally distributed and were compared with an unpaired *t* test. A *P* value less than 0.05 was considered significant.

## Results

### K/DEX Retains Lumbar Intrathecal Tracer at the Infusion Site while ISO Promotes Tracer Distribution along the Spine

**Fig. 1.**
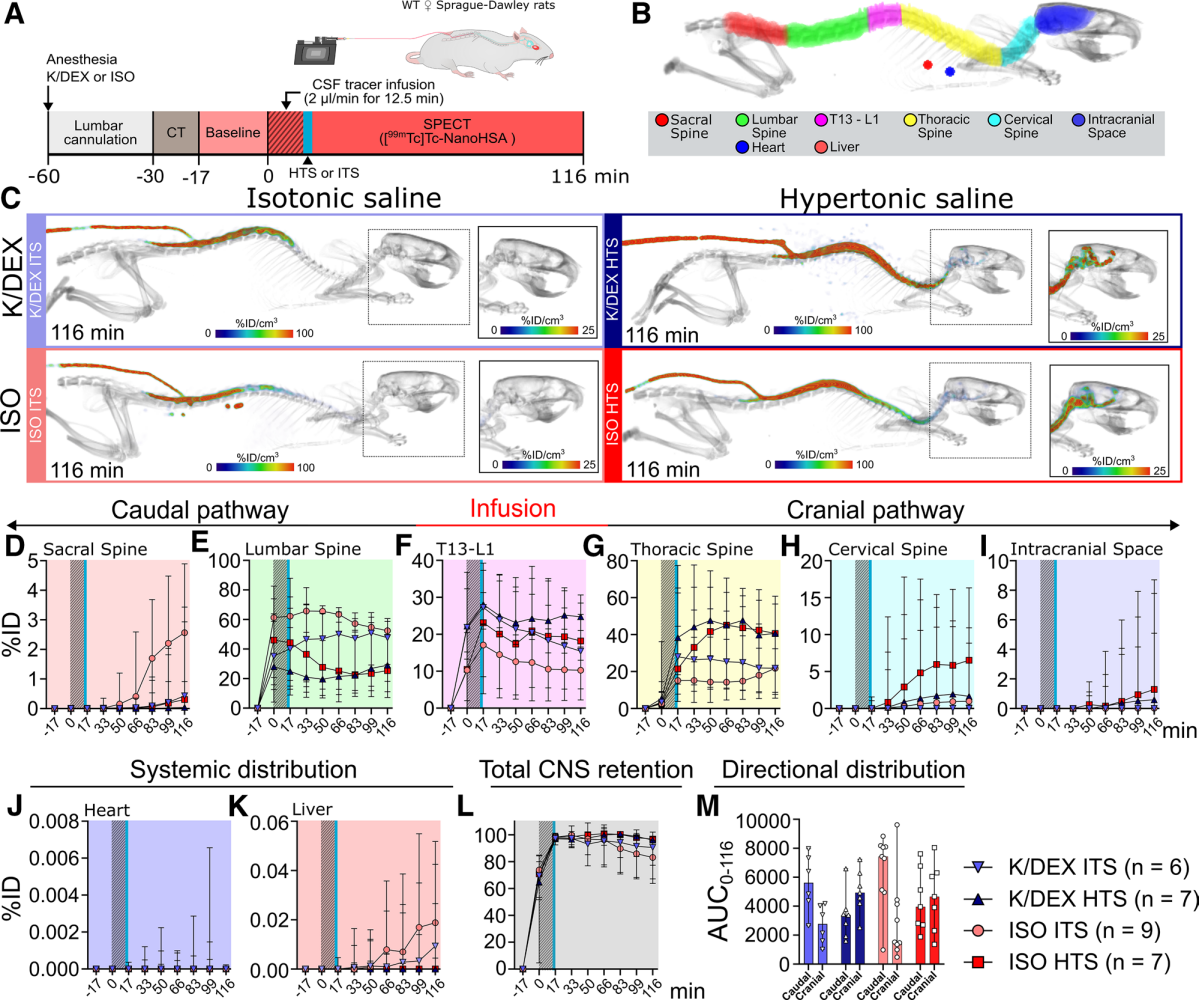
Distribution of lumbar intrathecal [^99m^Tc]Tc-NanoHSA (approximately 66.5 kDa) during K/DEX and ISO anesthesia in rats treated with either hypertonic saline (HTS) or isotonic saline (ITS). (*A*) Experimental setup. (*B*) Computed tomography (CT) with representative regions of interest (ROIs). (*C*) Representative single-photon emission CT (SPECT) images showing tracer distribution at 116 min. (*D–K*) Time–activity curves for the median percentage of injected dose (%ID) for (*D*) sacral spine, (*E*) lumbar spine, (*F*) T13–L1, (*G*) thoracic spine, (*H*) cervical spine, (*I*) intracranial space, (*J*) heart, and (*K*) liver. (*L*) Total CNS retention (*i.e.*, combined activity of all CNS ROIs). (*M*) The area under the time–activity curve between 0 and 116 min (AUC_0-116_) for the cranial and caudal [99mTc]Tc-NanoHSA distribution (*i.e.,* combined activity of the ROIs located cranial or caudal from the infusion point at T13–L1 level). *Light blue inverted triangles*, ITS (0.154 M NaCl; control), and *dark blue triangles*, HTS (1 M NaCl; 20 ml/kg), during K/DEX (ketamine, 100 mg/kg; dexmedetomidine, 0.5 mg/kg) anesthesia. *Light red circles*, ITS, and *dark red squares*, HTS, during ISO (1.5 to 2%) anesthesia. *Gray bars* represent tracer infusion (12.5 min; 2 µl/min). *Blue bars* represent HTS or ITS administration. Quantitative comparisons between groups are presented in tables 1 and 2 and Supplemental Digital Content tables S1 and S2 (https://links.lww.com/ALN/E274). *Error bars* represent range. [^99m^Tc]Tc-NanoHSA, technetium-99m radiolabeled human serum albumin nanocolloid; CNS, central nervous system; CSF, cerebrospinal fluid; K/DEX, ketamine–dexmedetomidine; ISO, isoflurane; WT, wild type.

To investigate whether the choice of anesthesia and HTS compared with ITS control influences the whole-body distribution of lumbar intrathecal [^99m^Tc]Tc-NanoHSA (66.5 kDa), we acquired dynamic SPECT images more than 116 min in female rats during K/DEX or ISO anesthesia (fig. 1A; Supplemental Digital Content video S1, https://links.lww.com/ALN/E275). Distribution of [^99m^Tc]Tc-NanoHSA was quantified for ROIs defined from the anatomical reference CT acquired before the SPECT (fig. 1B). The area under the time–activity curve between 0 and 116 min (AUC_0-116_), the maximum percentage of injected dose (C_max_), and time to maximum percentage of injected dose were calculated for each ROI. The time to first activity detected (arrival time) was calculated for ROIs other than the infusion site.

**Table 1.** Distribution of Lumbar Intrathecal [^99m^Tc]Tc-NanoHSA (66.5 kDa) during Ketamine–Dexmedetomidine (K/DEX; n = 6) *versus* Isoflurane (ISO; n = 9) Anesthesia in Rats Treated with Isotonic Saline (ITS)

Variable	ISO-ITS	K/DEX-ITS	Treatment Effect	*P* Value
C_max_ ICS	0.02 (0–5.1)	0	∞*	**0.0260**
AUC_0-116_ ICS	0.44 (0–186)	0	∞*	**0.0260**
AT ICS	99 (33–115.5)	u.d.	n.a.	
C_max_ CS	0.98 (0.04–16)	0.03 (0–0.48)	0.03	**0.0172**
AUC_0-116_ CS	53 (3–1,250)	1.05 (0–11)	0.02	**0.0076**
AT CS	33 (16.5–99)	90.75 (49.5–115.5)	57.75	0.0585
C_max_ TS	22 (6–78)	28 (11–45)	1.27	0.6070
AUC_0-116_ TS	1,519 (465–8,183)	2,762 (1,018–4,168)	1.82	0.4559
C_max_ T13–L1	18 (9–23)	27 (21–39)	1.49	**0.0016**
AUC_0-116_ T13–L1	1,488 (632–2,352)	2,643 (2,282–4,287)	1.78	**0.0016**
C_max_ LS	66 (13–88)	51 (30–70)	0.77	0.2721
AUC_0-116_ LS	7,409 (976–8,664)	5,611 (2,642–7,939)	0.76	0.2721
C_max_ SS	2.6 (0–4.9)	0.44 (0–3.4)	0.17	0.1510
AUC_0-116_ SS	103 (0–245)	11 (0–83)	0.11	0.0689
AT SS	0 (0–115.5)	41.25 (0–115.5)	41.25	0.2070
C_max_ liver	0.02 (0–0.04)	0.01 (0–0.03)	0.30	0.2845
AUC_0-116_ liver	0.72 (0–2.1)	0.24 (0–0.95)	0.33	0.1510
AT liver	49.50 (16.5–115.5)	41.25 (33–115.5)	8.25	0.5487
C_max_ CNS	100 (97–102)	99 (95–102)	0.99	0.7756
AUC_0-116_ CNS	11,181 (9,934–11,783)	11,185 (10,760–11,777)	1.00	0.9546
Cranial-to-caudal ratio	0.20 (0.05–9.9)	0.50 (0.13–1.6)	2.50	0.4559

Data are presented as median (minimum–maximum). Ratios between the treatment groups are presented for AUC_0-116_ and C_max_, while differences between medians are presented for AT. Statistical comparisons were carried out using two-tailed Mann–Whitney test. A *P* value less than 0.05 (presented with bold font) was considered significant. ISO, 1.5 to 2%; K/DEX, ketamine, 100 mg/kg, and dexmedetomidine, 0.5 mg/kg.

* The symbol ∞ refers to infinity as the denominator in the ratio is zero.

[^99m^Tc]Tc-NanoHSA, technetium-99m radiolabeled human serum albumin nanocolloid; AT, arrival time (minutes); AUC_0-116_, area under the time–activity curve between 0 and 116 min; C_max_, maximum percentage of injected dose; CNS, central nervous system; CS, cervical spine; ICS, intracranial space; ITS, 0.154 M NaCl; LS, lumbar spine; n.a., not available; SS, sacral spine; TS, thoracic spine; u.d., undetectable.

**Table 2.** Distribution of Lumbar Intrathecal [^99m^Tc]Tc-NanoHSA during Ketamine–Dexmedetomidine (K/DEX) Anesthesia in Rats Treated with Either Hypertonic Saline (HTS; n = 7) or Isotonic Saline (ITS; n = 6)

Variable	K/DEX-ITS	K/DEX-HTS	Treatment Effect	*P* Value
C_max_ ICS	0	0.6 (0–8.7)	∞*	**0.0047**
AUC_0-116_ ICS	0	22 (0–398)	∞*	**0.0047**
AT ICS	u.d.	66 (33–115.5)	n.a.	
C_max_ CS	0.03 (0–0.48)	2.2 (0.82–18)	72.3	**0.0012**
AUC_0-116_ CS	1.1 (0–11)	120 (18–1,269)	114	**0.0012**
AT CS	90.75 (49.5–115.5)	33 (16.5–66)	57.75	**0.0082**
C_max_ TS	28 (11–45)	48 (29–63)	1.69	**0.0140**
AUC_0-116_ TS	2,762 (1,018–4,168)	4,808 (2,514–5,564)	1.74	**0.0140**
C_max_ T13–L1	27 (21–39)	29 (23–38)	1.08	> 0.9999
AUC_0-116_ T13–L1	2,643 (2,282–4,287)	2,898 (2,623–4,330)	1.09	0.2343
C_max_ LS	51 (30–70)	39 (21–59)	0.75	0.1807
AUC_0-116_ LS	5,611 (2,642–7,939)	3,380 (1,600–6,552)	0.60	0.0734
C_max_ SS	0.44 (0–3.4)	0.04 (0–0.91)	0.09	0.6597
AUC_0-116_ SS	11 (0–83)	1.7 (0.06–35)	0.16	0.6282
AT SS	41.25 (0–115.5)	0 (0–115.5)	41.25	0.3106
C_max_ liver	0.005 (0–0.03)	0.0004 (0–0.01)	0.09	0.2471
AUC_0-116_ liver	0.2 (0–0.95)	0.009 (0–0.25)	0.04	0.1078
AT liver	41.25 (33–115.5)	82.50 (33–115.5)	41.25	0.3648
C_max_ CNS	99 (95–102)	101 (98–106)	1.01	0.2343
AUC_0-116_ CNS	11,185 (10,760–11,777)	11,526 (11,106–11,922)	1.03	0.1807
Cranial-to-caudal ratio	0.50 (0.13–1.6)	1.6 (0.38–4.5)	3.16	**0.0350**

Data are presented as median (minimum–maximum). Ratios between the treatment groups are presented for AUC_0-116_ and C_max_, while differences between medians are presented for AT. Statistical comparisons were carried out using two-tailed Mann–Whitney test. A *P* value less than 0.05 (presented with bold font) was considered significant. HTS, 1 M NaCl, 20 ml/kg; ITS, 0.154 M NaCl; K/DEX, ketamine, 100 mg/kg, and dexmedetomidine, 0.5 mg/kg.

* The symbol ∞ refers to infinity as the denominator in the ratio is zero.

[^99m^Tc]Tc-NanoHSA, technetium-99m radiolabeled human serum albumin nanocolloid; AT, arrival time (minutes); AUC_0-116_, area under the time–activity curve between 0 and 116 min; C_max_, maximum percentage of injected dose; CNS, central nervous system; CS, cervical spine; ICS, intracranial space; LS, lumbar spine; n.a., not available; SS, sacral spine; TS, thoracic spine; u.d., undetectable.

We first investigated how the distribution of lumbar intrathecal [^99m^Tc]Tc-NanoHSA is influenced by K/DEX and ISO anesthesia comparing the ITS control groups during these two anesthetic regimens. Compared to ISO, K/DEX resulted in a significantly higher tracer exposure at the site of infusion (T13–L1 AUC_0-116_ ratio, 1.78; *P* = 0.0016; table 1; fig. 1F), whereas ISO markedly increased the distribution of [^99m^Tc]Tc-NanoHSA along the spinal canal toward the intracranial space (cervical spine AUC_0-116_ ratio, 0.03; *P* = 0.0076; table 1; fig. 1H). In addition to the cranial spread, the tracer tended to spread caudally during ISO anesthesia even if this was not statistically significant (K/DEX to ISO AUC_0-116_ ratio in sacral spine, 0.11; *P* = 0.0689; table 1; fig. 1D). We found no significant differences between the choice of anesthesia for the thoracic spine, the lumbar spine, or the ROIs representing systemic circulation (*i.e.*, activities in ROIs placed within the heart and liver; table 1; fig. 1, E, G, J, and K). Moreover, there were no differences in CNS exposure (*i.e.*, activities in all CNS ROIs combined; table 1; fig. 1L) or when the ratio between the activities in ROIs cranial and caudal from the infusion point was calculated (cranial-to-caudal ratio; table 1; fig. 1M).

### Smaller Spinal Subarachnoid Space Volume during ISO Anesthesia: A Potential Mechanism Underlying Differences in CSF Flow by Anesthesia

**Fig. 2.**
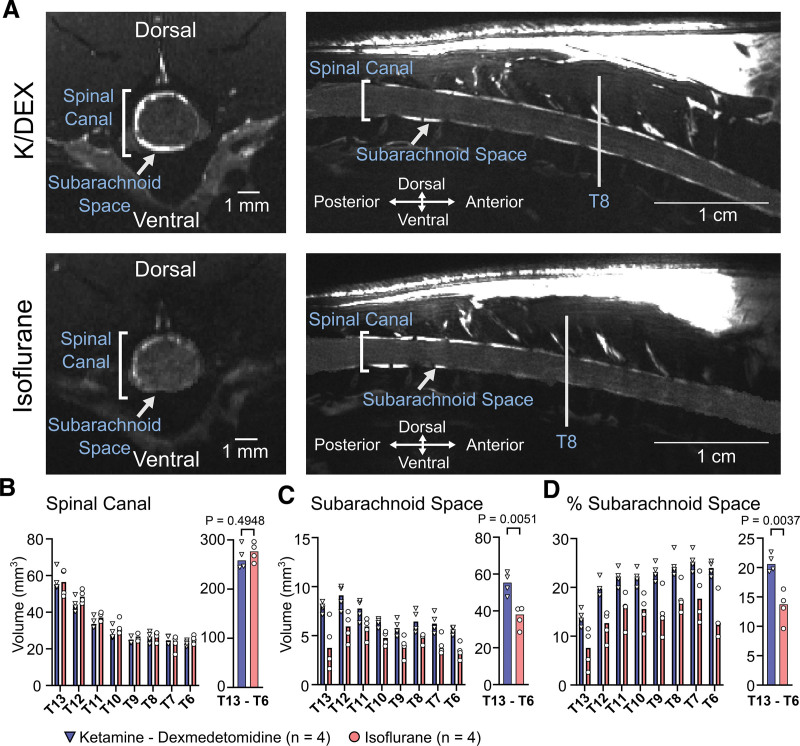
Magnetic resonance imaging of the spinal canal in rats during K/DEX and ISO anesthesia. (*A*) Representative visualization of cerebrospinal fluid (CSF) compartments using the high-resolution three-dimensional constructive interference steady state (3D-CISS) sequence. (*B*) Volume quantification for each spinal column T13–T6. (*C*) Subarachnoid space volume quantification for each spinal column level T13–T6. (*D*) Percentage subarachnoid space fraction for each spinal column. *Bars* represent median. Unpaired *t* tests were performed. K/DEX, ketamine, 100 mg/kg, and dexmedetomidine, 0.5 mg/kg; ISO, isoflurane 1.5–2%.

It has recently been suggested that a smaller CSF volume in the intracranial space during ISO compared to K/DEX anesthesia results in an increased spread of CM-administered tracers along the base of the brain and the spinal cord, reducing brain availability.^16,17^ To investigate whether the different anesthetic effects on tracer distributions after lumbar intrathecal administration could similarly be explained by changes in the spinal subarachnoid space volume, we performed structural MRI over the lower thoracic spinal canal from spinal segment T13 to T6. We found that the CSF filled subarachnoid space volume was 46% larger in rats anesthetized with K/DEX compared with ISO (*P* = 0.0051; fig. 2).

### HTS Increases Delivery of Lumbar Intrathecally Administered Tracer to the Brain during K/DEX but Not ISO Anesthesia

During K/DEX anesthesia, compared with the ITS control group, HTS increased the intracranial and spinal availability of the tracer at sites cranial to the infusion site with the largest difference in the intracranial space (AUC_0-116_ ratio, ∞; *P* = 0.0047) followed by the cervical spine (AUC_0-116_ ratio, 114; *P* = 0.0012) and the thoracic spine (AUC_0-116_ ratio, 1.74; *P* = 0.0140; table 2; fig. 1, C and G to I). Further, while no activity was detected in the intracranial space and close to none was detected in the cervical spine in the ITS group, HTS led to a C_max_ of approximately 1%ID in the intracranial space (*P* = 0.0047; table 2) and approximately 2%ID in the cervical spine (*P* = 0.0012; table 2). Compared with ITS, HTS also resulted in a significant difference in cranial-to-caudal ratio (AUC_0-116Cranial_/AUC_0-116Caudal_ ratio, 3.16; *P* = 0.0350; table 2; fig. 1M). No significant differences between HTS and ITS were observed at the infusion site (T13–L1), at the lumbar spine, at the sacral spine, in systemic circulation (*i.e.*, the ROIs placed over the heart and liver), or in the CNS during K/DEX anesthesia (table 2; fig. 1, D to F and J to L).

During ISO anesthesia, there were no differences in tracer exposure between the HTS and ITS groups in any of the ROIs, in total CNS exposure, or in the cranial-to-caudal ratio compared with ITS (Supplemental Digital Content table S1, https://links.lww.com/ALN/E274; fig. 1, C to M). Interestingly, the differences between the two anesthetic regimens disappeared with HTS as the cohorts did not differ in any of the evaluated areas during the different anesthetic regimens (Supplemental Digital Content table S2, https://links.lww.com/ALN/E274).

### Systemic HTS Retains Lumbar Intrathecal High-molecular-weight Tracer in the CNS in Awake Rats

**Fig. 3.**
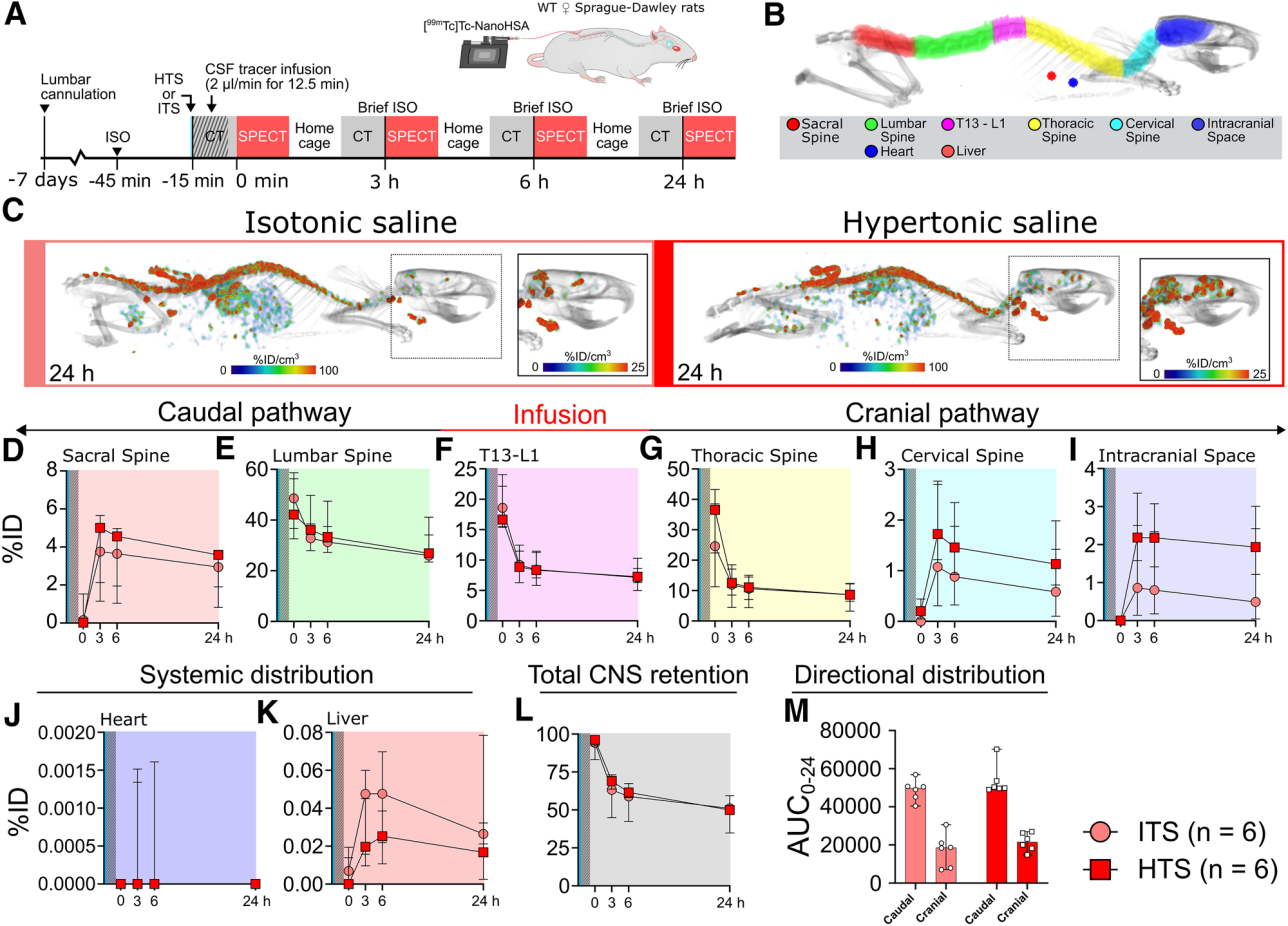
Distribution of lumbar intrathecal [^99m^Tc]Tc-NanoHSA (66.5 kDa) in awake rats scanned during brief ISO anesthesia and treated with either hypertonic saline (HTS) or isotonic saline (ITS). (*A*) Experimental setup. (*B*) Computed tomography (CT) with representative regions of interest (ROIs). (*C*) Representative single-photon emission CT (SPECT) images showing tracer distribution at 24 h. (*D–K*) Time–activity curves for the median percentage of injected dose (%ID) for (*D*) sacral spine, (*E*) lumbar spine, (*F*) T13–L1, (*G*) thoracic spine, (*H*) cervical spine, (*I*) intracranial space, (*J*) heart, and (*K*) liver. (*L*) Total CNS retention (*i.e.*, combined activity of all CNS ROIs). (*M*) The area under the time–activity curve between 0 and 24 h (AUC_0-24_) for the cranial and caudal [99mTc]Tc-NanoHSA distribution (*i.e.*, combined activity of the ROIs located cranial or caudal from the infusion point at T13–L1 level). *Light red circles* depict the median activity under ITS (0.154 M NaCl; control), and *dark red squares* depict the median activity under HTS (1 M NaCl; 20 ml/kg). *Gray bars* represent tracer infusion (12.5 min; 2 µl/min). *Blue lines* represent HTS or ITS administration. Quantitative comparisons between groups are presented in table 3. *Error bars* represent range. [^99m^Tc]Tc-NanoHSA, technetium-99m radiolabeled human serum albumin nanocolloid; CNS, central nervous system; CSF, cerebrospinal fluid; ISO, isoflurane; WT, wild type.

After demonstrating a strong effect of HTS on tracer distribution during K/DEX anesthesia and a large influence of anesthesia on tracer distribution, we investigated tracer dynamics beyond 116 min. To investigate the distribution of lumbar infused [^99m^Tc]Tc-NanoHSA at more than 24 hours, we performed CT-SPECT acquisitions at baseline and 3, 6, and 24 h after infusion. The rats were scanned during brief ISO anesthesia and stayed awake in their home cage between the scans (fig. 3).

**Table 3.** Distribution of Lumbar Intrathecal [^99m^Tc]Tc-NanoHSA (66.5 kDa) in Awake Rats Scanned during Brief Isoflurane Anesthesia and Treated with Either Hypertonic Saline (HTS; n = 6) or Isotonic Saline (ITS; n = 6)

Variable	ITS	HTS	Treatment Effect	*P* Value
C_max_ ICS	0.86 (0.18–3.4)	2.3 (1.6–2.5)	2.61	0.1797
AUC_0-24_ ICS	15 (2.7–69)	48 (33–53)	3.10	0.1320
3 h ICS	0.86 (0.14–3.4)	2.2 (1.6–2.5)	2.53	0.1797
C_max_ CS	1.1 (0.35–2.8)	1.7 (1.2–2.7)	1.60	0.1797
AUC_0-24_ CS	18 (6–51)	31 (21–41)	1.77	0.1797
3 h CS	1.08 (0.31–2.8)	1.7 (1.2–2.7)	1.60	0.1797
C_max_ TS	25 (11–43)	37 (22–39)	1.49	0.5887
AUC_0-24_ TS	16,292 (6,481–23,375)	16,797 (11,550–21,981)	1.03	0.6991
3 h TS	12 (4.5–18)	13 (8.5–17)	1.05	0.6991
C_max_ T13–L1	19 (15–24)	17 (16–22)	0.90	> 0.9999
AUC_0-24_ T13–L1	12,460 (8,881–16,693)	12,555 (10,908–15,211)	1.01	0.8182
3 h T13–L1	9.2 (6.3–12)	8.9 (8.2–11)	0.96	0.8182
C_max_ LS	49 (33–59)	42 (37–56)	0.87	0.6991
AUC_0-24_ LS	44,614 (37,764–52,583)	45,823 (43,440–66,120)	1.03	0.5887
3 h LS	33 (28–38)	36 (34–50)	1.10	0.0931
C_max_ SS	3.8 (2.1–4.8)	5 (1.1–5.7)	1.31	0.3095
AUC_0-24_ SS	4,645 (2,642–5,628)	5,710 (1,310–6,151)	1.23	0.3939
3 h SS	3.8 (2.1–4.8)	5 (1.1–5.7)	1.33	0.3095
C_max_ liver	0.05 (0.02–0.07)	0.03 (0.02–0.05)	0.50	**0.0260**
AUC_0-24_ liver	55 (27–85)	27 (15–46)	0.49	**0.0260**
3 h liver	0.05 (0.01–0.06)	0.02 (0.02–0.05)	0.41	0.1320
C_max_ CNS	94 (83–97)	96 (92–97)	1.02	0.1320
AUC_0-24_ CNS	1,415 (1,018–1,434)	1,450 (1,379–1,603)	1.02	0.3095
3 h CNS	63 (45–67)	69 (64–73)	1.09	**0.0411**
Cranial-to-caudal ratio	0.35 (0.16–0.76)	0.43 (0.21–0.54)	1.25	0.3939

Data are presented as median (minimum–maximum). Ratios between the treatment groups are presented for AUC_0-24_ and C_max_, while differences between medians are presented for AT. Statistical comparisons were carried out using two-tailed Mann–Whitney test. A *P* value less than 0.05 (presented with bold font) was considered significant. HTS, 1 M NaCl, 20 ml/kg; ITS, 0.154 M NaCl.

[^99m^Tc]Tc-NanoHSA, technetium-99m radiolabeled human serum albumin nanocolloid; AUC_0-24_, area under the time–activity curve between 0 and 24 h; C_max_, maximum percentage of injected dose; CNS, central nervous system; CS, cervical spine; ICS, intracranial space; LS, lumbar spine; SS, sacral spine; TS, thoracic spine.

Here, we found that HTS significantly decreased the egress of the tracer from the CNS as reflected by lower activity in the liver ROI (area under the time–activity curve between 0 and 24 h ratio, 0.49; *P* = 0.0260) and a significantly higher activity in the CNS 3 h after tracer infusion (3 h CNS ratio, 1.09; *P* = 0.0411) compared with ITS (table 3; fig. 3, J to L). For the remaining ROIs assessed, we found no significant change in tracer distribution (table 3; fig. 3).

## Discussion

We found that lumbar intrathecal radiotracer was retained at the site of administration during K/DEX anesthesia, whereas ISO resulted in tracer distribution toward the cervical spine and intracranial space. The increased tracer spread away from the infusion site during ISO anesthesia may be explained by the smaller CSF-filled spinal subarachnoid space during ISO anesthesia compared with K/DEX as visualized with MRI. Systemic HTS increased tracer availability in the intracranial space and cervical and thoracic spine during K/DEX anesthesia. Indeed, the tracer availability in the intracranial space was increased from zero to about 1% of injected dose. Systemic HTS did not further increase the intracranial availability during ISO anesthesia. In awake rats, systemic HTS prolonged tracer retention in the CNS.

Previous studies in rats have shown that K/DEX anesthesia and other anesthetic regimens containing α_2_-agonists, *i.e.*, dexmedetomidine and xylazine, result in a higher brain availability of CM-infused tracers, likely due to enlarged periarterial spaces,^16–18^ interstitial space size,^29^ and increased slow-wave activity in electroencephalogram.^19,30^ In contrast, ISO anesthesia promotes fast egress of tracers from the CNS to systemic circulation, partly due to a shrinkage in CSF space volume.^16–18^ These findings have been interpreted to mean that anesthetic regimens containing α_2_-adrenergic agonists enhance brain uptake of CSF-administered drugs regardless of the administration level.^16–20^ However, the current study shows that K/DEX anesthesia promotes tracer retention at the lumbar infusion site instead of promoting delivery to the brain. These findings could have direct clinical implications for patient groups anesthetized for lumbar spinal drug delivery, such as pediatric patients.

Systemic HTS is used clinically for elevated intracranial pressure.^31^ Increased plasma osmolarity draws water from the brain parenchyma to the systemic circulation, effectively shrinking the brain and decreasing intracranial pressure.^24,31,32^ Interestingly, several preclinical studies have recently shown that a part of the withdrawn water is replaced by CSF flowing into the brain from the perivascular space even if the net effect is a reduction in the fluid content of the brain. These findings have spurred the idea that systemic HTS could be used to enhance the brain delivery of CSF-administered drugs. Indeed, systemic administration of 40 mOsm/kg HTS enhanced the brain delivery of nanoparticles,^21^ small-molecular tracers (489 Da),^22^ and immunoglobulins (immunoglobulin G; 150 kDa)^24^ after CM delivery during K/DEX or ketamine–xylazine anesthesia.

We found that during K/DEX anesthesia, systemic HTS enhanced the cranial delivery of a spinal large-molecule tracer, whereas during ISO anesthesia, the tracer distribution was similar in the HTS and ITS groups. Indeed, the poor intracranial availability of the tracer duringK/DEX anesthesia could be augmented with HTS to match the effect of ISO anesthesia. Interestingly, the total CNS retention of the tracer was similar between the HTS- and ITS-treated rats during K/DEX anesthesia, suggesting that HTS did not affect efflux from the CNS despite the change in distribution. Taken together, our findings suggest that a part of the CSF flowing into the fluid-deprived brain after an HTS bolus comes from the spinal canal. Furthermore, K/DEX is not an appropriate anesthetic choice for administering spinal drugs targeting the brain unless boosted with HTS. However, based on our current and previous findings,^33^ anesthetic regimens containing α_2_-adrenergic agonists are particularly well-suited to enhance retention at the spinal administration level.

Lumbar spinal administration of therapeutics is a procedure that often is performed awake rather than during anesthesia in adult patients. A previous study demonstrated that systemic HTS increased the spinal cord delivery of lumbar spinal morphine (285 Da) in awake rats.^27^ While we could not differentiate between the availability of the tracer in the parenchyma and the subarachnoid space due to the limited resolution of SPECT imaging, it is possible that the longer tracer availability in the CNS is due to parenchymal uptake and longer clearance as a result. Although systemic HTS seemed to increase intracranial availability of [^99m^Tc]Tc-nanoHSA in awake rats (fig. 3I), the difference was not significant due to large variance (table 3). Enhanced tracer retention in the CNS induced by systemic HTS in the awake state could be used to prolong the duration of action of drugs, such as leptomeningeal chemotherapy, that exhibit limited time at therapeutic concentrations in the CNS due to rapid clearance.^7^

Our study expands on the limited information regarding intracranial distribution of drugs administered into the CSF space at the lumbar level, which is the frequently used route of intrathecal administration in clinical practice. While one clinical study investigated how the administration volume and external pulsations over the chest or abdomen influence cranial delivery,^10^ previous preclinical studies have mainly focused on lumbar administration for spinal targets^27,33^ and CM^16–18,21–24,34^or intraventricular^25^ administration for brain targets. The use of *in vivo* whole-body SPECT imaging allowed us to map the tracer distribution in multiple directions and at different time points, enabling the investigation of pharmacokinetic parameters. However, the limited spatial resolution of SPECT did not allow us to differentiate between compartments, such as the brain parenchyma and the CSF space. For example, the higher intracranial tracer availability during ISO anesthesia does not necessarily reflect effective brain penetrance. Further, the spinal catheter placement prevented us from differentiating between tracer left in the tunneled catheter from the real signal in the lumbar spine, hence overestimating tracer activity in the caudal direction. Finally, in this study, only female rats were included as they are more homogenous in size and weight compared to male rats and are hence a more suitable model for the study questions. Although this might limit the interpretation of our findings in male rats and humans, we believe that the potential effect of sex is limited compared to the effect of anesthetics and hypertonic solutions, which are the strongest known pharmacologic agents influencing CSF flow. A previous study investigated the influence of biological sex on glymphatic influx between anesthetized female and male mice and found no differences.^26^ Indeed, in that study, anesthetics were found to influence CSF influx in the same way in both sexes—or, if there were any differences, the anesthetic effects overcame the influence of sex. This finding suggests that major sex differences might not exist between female and male rats either. Finally, most preclinical research on the CSF flow has been conducted in male rodents, and therefore our study expands understanding of CSF flow in female rodents.

### Conclusions

We demonstrated that anesthesia has a pivotal role in the spread of intrathecally administered drugs at the spinal and intracranial levels. Further, we showed that, in awake and K/DEX anesthetized rats, systemic HTS could prolong and elevate the CNS availability of the tracer, respectively. Given that the tested interventions—anesthetics and systemic HTS—are clinically used and available interventions, we propose that our findings should be subjected to clinical trials.

## Acknowledgments

The Radiopharmaceutical Chemistry service of the Helsinki *In Vivo* Animal Imaging Platform of the Helsinki Institute of Life Science (Helsinki, Finland) is acknowledged for radiotracer production. The SPECT/CT imaging services of Biocentre Finland and the Helsinki *In Vivo* Animal Imaging Platform of Helsinki Institute of Life Science (Helsinki, Finland) as well as the Panum Preclinical MRI Core Facility at the Faculty of Health and Medical Sciences, University of Copenhagen (Copenhagen, Denmark), are acknowledged for animal imaging.

## Research Support

This work was supported by Academy of Finland (grant No. 350371; Helsinki, Finland), Finnish Medical Foundation (Helsinki, Finland), Paulo Foundation (Espoo, Finland), Sigrid Jusélius Foundation (Helsinki, Finland), Emil Aaltonen Foundation (Tampere, Finland), Finnish Foundation for Drug Research (Helsinki, Finland), Lundbeck Foundation (Copenhagen, Denmark), Novo Nordisk Foundation (Hellerup, Denmark), Acta Anaesthesiologica Scandinavica Foundation (Copenhagen, Denmark), University of Helsinki Research Funds (Helsinki, Finland), University Doctoral School, University of Helsinki (Helsinki, Finland), and Helsinki One Health (Helsinki, Finland).

## Competing Interests

Dr. Lilius and Dr. Nedergaard declare the possession of a U.S. patent with the title “Glymphatic Delivery by Manipulating Plasma Osmolarity” (patent No. US-20220280423-A1). The other authors declare no competing interests.

## Supplemental Digital Content

Supplemental Methods, Figure, Tables, https://links.lww.com/ALN/E274

Supplemental Video, https://links.lww.com/ALN/E275

## Supplementary Material
