# Supplementary material for: General Anesthesia and Systemic Hyperosmolality Modulate Lumbar Intrathecal Drug Distribution in Female Rats
Source: Anesthesiology. 2025 Oct 9;144(2):390–401. doi: 10.1097/ALN.0000000000005794 (PMC12777593; doi:10.1097/ALN.0000000000005794)
Supplement: Supplementary file 1 [file aln-144-390-s001.pdf]

Supplementary Digital Content: General anesthesia and systemic hyperosmolality modulate lumbar intrathecal drug distribution in female rats

Niklas Daniel Åke Persson, M.Sc.[1,2,3], Terhi J. Lohela, M.D., Ph.D,[1,3,4], Jenni E. Anttila, Ph.D. (Pharm.)[1,3], Jenni Mäkelä, B.M.[1,2], Marko Rosenholm, Ph.D. (Pharm)[3,5], Martta Peltoniemi, M.Sc.[1,3,6], Sonja C. Jalonen, M.Sc.[1,3], J. Arturo García-Horsman, Ph.D.[3], Mirkka Sarparanta, Ph.D.[6], Maiken Nedergaard, M.D., D.M.Sc[5,7], Tomi Rantamäki,Ph.D. (Pharm.)[3,8], Tuomas O. Lilius, M.D., Ph.D.[1,2,3,9]

1. *Individualized Drug Therapy Research Program, Faculty of Medicine, University of Helsinki, Helsinki, Finland*
2. *Department of Pharmacology, Faculty of Medicine, University of Helsinki, Helsinki, Finland*
3. *Division of Pharmacology and Pharmacotherapy, Drug Research Program, Faculty of Pharmacy, University of Helsinki, Helsinki, Finland*
4. *Department of Anaesthesiology, Intensive Care and Pain Medicine, HUS Helsinki University Hospital and University of Helsinki, Helsinki, Finland*
5. *Center for Translational Neuromedicine, Faculty of Health and Medical Sciences, University of Copenhagen, Copenhagen, Denmark*
6. *Department of Chemistry, Radiochemistry, University of Helsinki, Helsinki, Finland*
7. *Center for Translational Neuromedicine, University of Rochester Medical Center, Rochester, NY, USA*
8. *SleepWell Research Program, Faculty of Medicine, University of Helsinki, Helsinki, Finland*
9. *Department of Emergency Medicine and Services, HUS Helsinki University Hospital and University of Helsinki, Helsinki, Finland*

Supplementary Methods 1

Lumbar catheterization

Rats were anesthetized with either a mixture of K/DEX containing ketamine (100 mg/kg, Ketaminol vet 50 mg/ml or 100 mg/ml, Intervet, Boxmeer, The Netherlands) and dexmedetomidine (0.5 mg/kg, Dexdomitor 0.5 mg/ml, Orion Pharma, Espoo, Finland) by subcutaneous injection (2 ml/kg) or by ISO (isoflurane; 3% induction, 1.5–2% maintenance, Vetflurane, Virbac, Carros, France). After loss of toe-pinch reflex, a cannula constituted of PE10 tubing (25 cm, PE10, 0.28 mm i.d. × 0.61 mm o.d., Clay Adams INTRAMEDIC Polyethylene, BD, Franklin Lakes, NJ), was implanted into the lumbar intrathecal subarachnoid space as previously described.[1] Briefly, the fur was shaved above the L6 vertebra, and a small incision was made to uncover the subcutaneous space. The spinal catheter was inserted in the subarachnoid space between the L5 and L6 vertebrae with a guide cannula (20-gauge needle). Correct localization of guide cannula in the subarachnoid space was confirmed by a slight flick of the paw or tail. The catheter was carefully tunneled in the subarachnoid space until the level of the caudal ribs (T13). The guide cannula was carefully removed, a metal wire was inserted in the catheter and a bubble was made with a cautery pen before the catheter was attached to paravertebral muscles with 4–0 sutures to avoid dislocation of the catheter. For experiments using dynamic SPECT, the catheter was cut with 8 cm dead volume and left exposed at the back of the rat and filled with saline before the tip was closed by cauterization.

In the 24-hours SPECT experiments, the rest of the 25-cm long catheter was tunneled subcutaneously, revealed from the neck, and sutured to paravertebral muscles of the neck. The catheter was flushed and filled with saline, and the tip was closed by cauterization.

After confirming that there were no leaks from the catheter, cutaneous openings were closed with 4–0 sutures. Carprofen (5 mg/kg s.c., Rimadyl, Zoetis, Lincoln, NE, USA) was administered perioperatively and postoperatively for two days to rats recovering from anesthesia. Correct placement of chronically placed catheters was confirmed by injecting 15 µl lidocaine (20 mg/ml, Lidocain®, Orion Pharma, Espoo, Finland) flushed down by 20 µl of saline 1-2 days before SPECT experiments. Short paralysis of at least one hind paw was considered as correct placement.

Tracer preparation and quality control

The ^{99}Mo/[^{99m}Tc]Tc-generator (Ultra-TechneKow FM 2.15-4.30 GBq, obtained either via Curium Netherlands, B.V., Petten, Holland or GE HealthCare, Helsinki, Finland) was eluted with 5 ml sterile evacuated vial according to the manufacturer instructions using 4 ml of sterile, pyrogen-free 0.9% NaCl. Approximately 2 ml of the eluted [^{99m}Tc]TcO_4^- was added to the reaction vial (ROTOP-NanoHSA 0.5 mg, ROTOP Pharmaka GmbH, Dresden, Germany). The albumin concentration of the product was 0.29 ± 0.04 mg/ml. The vial was carefully mixed and let to react at room temperature for 10 minutes. The quality control (QC) was done with radio-thin layer chromatography (TLC) method using normal phase silica gel TLC plates (Silica gel on Al foil, 0.75 ml/g pore volume with fluorescence indicator at 254 nm, Sigma Aldrich, Saint Louis, MO, USA) and acetone as the eluent. After elution, the TLC plate was cut in half and radioactivity in the bottom and top pieces was counted with an automated gamma counter (Hidex, Turku, Finland). The retardation factors (R_f) used were R_f = 0 for [^{99m}Tc]Tc-nanoHSA and R_f = 1 for [^{99m}Tc]TcO_4^-. Radiochemical purity (RCP) was calculated from equation 1.

$$\mathrm{RCP} = \frac{\text{Activity at } R_f = 0}{\text{Total radioactivity on plate}} \times 100\%$$

The radiochemical purity of all tracer batches exceeded 95%. Additionally, refractometry (PTR2a, Index Instruments Ltd., UK) was used to confirm the isobaricity of the radiotracer with CSF (1.005–1.007 g/ml and 1.004–1.007 g/ml respectively).[2]

For *in vitro* radiolabel stability assays, approximately 20 MBq of tracer solution was dispensed into 1 ml rat brain homogenate (1:1 homogenized whole brain: 1 ×PBS, pH 7.4) in a 5-ml microtube. The mixture was incubated at 37 °C with shaking at 300 rpm and samples (100 µl) were withdrawn at designated time points (30, 60, 240, 480, and 1440 minutes). The samples were diluted with 200 µl of ice-cold 1×PBS, pH 7.4, and filtered off with a centrifugal filter. A 2 µl sample of the filtrate was spotted on a glass microfiber chromatographic paper iTLC-SA (polysilic acid, Agilent Technologies, Santa Clara, CA, USA), and developed with 0.1 M sodium citrate. After the TLC-plates were let dry, they were exposed to a digital autoradiography plate (Fujifilm, BAS-IP-TR imaging plates 20 × 25 cm) for 30 minutes. The plates were scanned with a photostimulated luminescence scanner FLA 5100 (Fujifilm, Tokyo, Japan) using Image reader FLA-5000 series V 1.0 computer program. The images were analyzed with the Aida Image Analyzer V 4.0 Program, taking R_f 0.9–1.0 for free [^{99m}Tc]Tc^- and R_f 0.0–0.1 for [^{99m}Tc]Tc-nanoHSA. The tracer showed excellent radiolabel stability in brain homogenate with percentage of intact radiolabel staying above 95% over 24 hours (n = 3) (Supplementary Fig. 1).

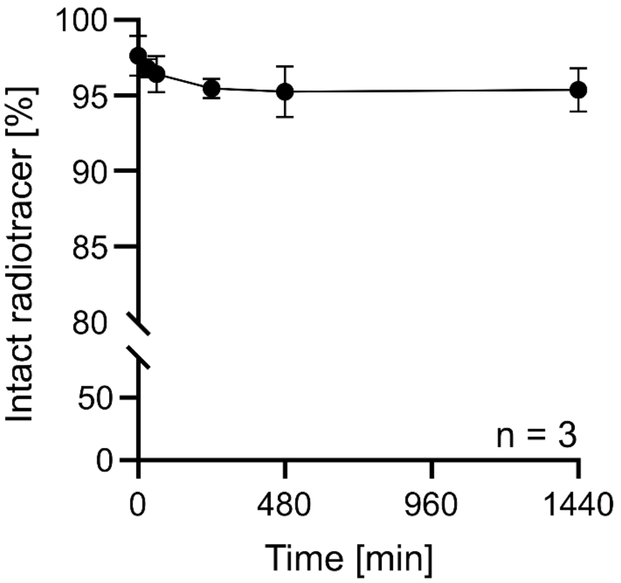

Supplementary Fig. 1. In vitro radiolabel stability of [99mTc]Tc-nanoHSA in rat brain homogenate. *The percentage of intact radiolabel was quantified 30, 60, 240, 480, and 1440 minutes after formulation (i.e. up to 24h) using radio-thin layer chromatography (TLC). Error bars represent standard deviation.*

Single-photon emission computed tomography imaging

For dynamic SPECT acquisitions, anesthetized rats with a lumbar cannula received an intraperitoneal catheter attached to a 5 ml syringe filled with 20 ml/kg hypertonic saline (HTS, 1 M NaCl) or isotonic saline (ITS, 0.154 M NaCl, control group) and were placed directly in a prone position within a small animal SPECT/CT system (Bioscan NanoSPECT/CT, Mediso, Budapest, Hungary). Body temperature was maintained utilizing a built-in heating system in the scanner bed and was monitored together with respiratory rate in between SPECT frames. A computed tomography (CT) reference image was collected with nine consecutive dynamic whole-body SPECT acquisitions over 116 minutes. [^{99m}Tc]Tc-nanoHSA was infused to the lumbar intrathecal (i.t.) space (25 µl at a rate of 2 µl/min) utilizing a Hamilton Gastight 1700 microsyringe (Hamilton-

Bonaduz, Bonaduz, Switzerland) attached to a Harvard PHD Ultra pump (Harvard apparatus, Holliston, Massachusetts, USA) starting at the beginning of second frame. Hypertonic saline (HTS, 1 M NaCl, 20 ml/kg equivalent to 40 mOsm/kg) or isotonic saline (ITS, 0.154 M NaCl, control group) was injected intraperitoneally (i.p.) over 2 minutes directly after CSF tracer infusion. SPECT images were acquired at a temporal resolution of 16.5 minutes, FOV $24 \times 24 \times 200$ mm. The following groups were studied K/DEX-ITS (n = 6), K/DEX-HTS (n = 7), ISO-ITS (n = 9) and ISO-HTS (n = 7). Sample size was estimated based on previous studies on the influence of anesthetics or hypertonic solutions on CSF distribution imaged with SPECT in rats.[3-6] While the order of the anesthetic was not randomized, the order of the hypertonic intervention was random with both ITS and HTS treated animals imaged during each experimental day. Experimental data were excluded from further analysis due to failed tracer infusions (n = 6) or failed image acquisition (n = 1). No further animals or data points were excluded during the analysis.

For 24-hours SPECT acquisitions, rats with a chronic catheter were anesthetized with ISO and sutures over the L4-L6 were removed to expose the catheter. HTS or ITS were injected i.p. and rats were immediately placed in a prone position within the NanoSPECT/CT system. A CT reference image was collected with one consecutive full body SPECT acquisition. [^{99m}Tc]Tc-NanoHSA was infused to the lumbar i.t. space (25 µl at a rate 2 µl/min) concurrently with the first CT acquisition. After SPECT acquisition finished, the catheter was closed and placed under the skin before the skin was closed with two sutures and the rat was let to wake up in the home cage. CT/SPECT acquisition was repeated under ISO anesthesia 3, 6, and 24 hours after tracer infusion. The following groups were studied for 24-hours; ITS (n = 6) and HTS (n = 6). Sample size was estimated

based on previous studies on the influence of anesthetics or hypertonic solutions on CSF distribution imaged with SPECT in rats.[3-6] The order of the hypertonic intervention was random with both ITS and HTS treated animals imaged during each experimental day. Experimental data were excluded from further analysis due to failed tracer infusions (n = 2). No further animals or data points were excluded during the analysis.

Magnetic resonance imaging

Anesthetized rats under K/DEX (n = 4) or ISO (n = 4) were placed in prone position in an MR-compatible stereotactic holder and secured with ear bars (Bruker BioSpin; Bruker, Ettlingen, Germany). Body temperature was maintained at 37 ± 0.5°C utilizing a hot air system and monitored along with the respiratory rate by an MR-compatible remote-monitoring system (SA Instruments, Stony Brook, NY). Imaging was performed using a 9.4 T animal scanner (BioSpec 94/30 USR, Paravision 6.0.1 software; Bruker) equipped with 240 mT/m gradient coil (BGA-12S; Bruker) an 86 mm volume RF-transmit coil and a surface RF-receive coil (Bruker). T2-weighted structural imaging was acquired using three-dimensional constructive interference steady state (3D-CISS). Every 3D-CISS image was calculated as a maximum intensity projection from four realigned 3D-TrueFISP volumes with four orthogonal phase-encoding directions e.g., 90º, 270º, 180º and 360º (echo time [TE]: 2.85 ms, repetition time [TR]: 5.700 ms, flip angle [FA] 50º, matrix: 480 × 256 × 128, FOV 48 × 25.6 × 12.8 mm). The order of the anesthetic was randomized.

Single-photon emission computed tomography data analysis

Acquired images were reconstructed by Nucline acquisition software (Mediso, Budapest, Hungary). Regions of interest (ROIs) were determined utilizing ITK-SNAP.[7] Our ROIs

included intracranial, cervical spine, thoracic spine (except T13), T13–L1, lumbar spine (except L1), sacral spine, liver, and heart. In addition, we combined ROIs to calculate the total CNS, the cranial pathway and the caudal pathway and calculated the cranial-to-caudal pathway ratio. The total injected activity was measured by applying a whole-body segment at the first frame after all tracer infusion. The percentage of injected dose (%ID) in each ROI was calculated utilizing MATLAB R2022a (MathWorks, USA). Data acquisitions were performed unblinded while ROIs were drawn blinded.

Magnetic resonance imaging data analysis

Motion and bias field correction was applied to each of the four 3D-TrueFISP volumes collected for each animal. ANTs (Advanced Normalization Tools, version 2.3.4) was utilized for the motion correction.[8] An in-house software was used to correct ventral–dorsal second-order bias field arising from the use of a surface receiver coil placed at the dorsal side of the spine, and N4 was used for local bias filed correction.[8] A 3D-CISS image was calculated as a maximum intensity projection from the four realigned 3D-TrueFISP. ROIs was drawn over each spinal column and corresponding subarachnoid space ranging from T13-T6 utilizing the ITK-SNAP software. The percentage of subarachnoid space volume of the spinal canal was calculated for each column individually defined as the $\frac{\text{subarachnoid space volume}}{\text{total column volume}} \times 100\%$. Data acquisitions and analyses were performed unblinded.

Supplementary Tables

Table S1. Distribution of lumbar intrathecal [^{99m}Tc]Tc-NanoHSA (66.5 kDa) under isoflurane (ISO) anesthesia in rats treated with either hypertonic (HTS, n = 7) or isotonic saline (ITS, n = 9).

Variable	ISO-ITS	ISO-HTS	Treatment Effect	P-value
C_{max} ICS	0.02 (0–5.1)	1.3 (0–7.8)	65	0.1809
AUC ICS	0.44 (0–186)	41 (0–283)	93.2	0.1809
AT ICS	99 (33–115.5)	33 (33–115.5)	66	0.2351
C_{max} CS	0.98 (0.04–16)	6.8 (1.1–12)	6.94	0.2105
AUC CS	53 (3–1250)	411 (73–909)	7.75	0.2105
AT CS	33 (16.5–99)	33 (16.5–33)	0	0.4352
C_{max} TS	22 (6–78)	45 (14–66)	2.05	0.1416
AUC TS	1519 (465–8183)	3959 (1242–6959)	2.61	0.1416
C_{max} Th13-L1	18 (9–23)	24 (4–34)	1.30	0.1738
AUC Th13-L1	1488 (632–2352)	2350 (314–3405)	1.58	0.1142
C_{max} LS	66 (13–88)	46 (26–75)	0.70	0.5360
AUC LS	7409 (976–8664)	3930 (1881–7604)	0.53	0.0907
C_{max} SS	2.6 (0–4.9)	0.3 (0.002–2.9)	0.12	0.0907
AUC SS	103 (0–245)	9.5 (0.04–82)	0.09	0.0907
AT SS	0 (0–115.5)	0 (0–115.5)	0	0.8678
C_{max} Liver	0.02 (0–0.04)	0.002 (0–0.05)	0.13	0.2192
AUC Liver	0.72 (0–2.1)	0.04 (0–2.6)	0.07	0.1483

AT Liver	49.50 (16.5–115.5)	82.50 (16.5–115.5)	33	0.1902
C_{max} CNS	100 (97–102)	102 (96–109)	1.01	0.1416
AUC CNS	11181 (9934–11783)	11484 (9992–12200)	1.03	0.6065
Cranial-to-Caudal ratio	0.20 (0.05–9.9)	1.2 (0.18–4.3)	6	0.1416

Data are presented as median (min-max). Ratios between the treatment groups are presented for AUC and C_{max}, while differences between medians are presented for AT. Statistical comparisons were carried out using two-tailed Mann–Whitney test. AT, arrival time (minutes); C_{max}, maximum activity; AUC, area under the time-activity curve; ICS, intracranial space; CS, cervical spine; TS, thoracic spine; LS, lumbar spine; SS, sacral spine. ISO, isoflurane 1.5-2%. ITS, 0.154 M NaCl. HTS, 1 M NaCl, 20 ml/kg.

Table S2. Distribution of lumbar intrathecal [^{99m}Tc]Tc-NanoHSA (66.5 kDa) under ketamine-dexmedetomidine (K/DEX, n = 7) and isoflurane (ISO, n = 7) anesthesia in rats treated with hypertonic (HTS).

Variable	ISO-HTS	KDEX-HTS	Treatment Effect	P-value
C_{max} ICS	1.3 (0–7.8)	0.6 (0–8.7)	0.46	0.6480
AUC ICS	41 (0–283)	22 (0–398)	0.54	0.8368
AT ICS	33 (33–115.5)	66 (33–115.5)	33	0.3852
C_{max} CS	6.8 (1.1–12)	2.2 (0.82–18)	0,32	0.7104
AUC CS	411 (73–909)	120 (18–1269)	0.29	0.7104
AT CS	33 (16.5–33)	33 (16.5–66)	0	0.4493
C_{max} TS	45 (14–66)	48 (29–63)	1.07	>0.9999
AUC TS	3959 (1242–6959)	4808 (2514–5564)	1.21	0.8048
C_{max} Th13-L1	24 (4–34)	29 (23–38)	1.21	0.2593
AUC Th13-L1	2350 (314–3405)	2898 (2623–4330)	1.23	0.0530
C_{max} LS	46 (26–75)	39 (21–59)	0.85	0.3176
AUC LS	3930 (1881–7604)	3380 (1600–6552)	0.86	0.4557
C_{max} SS	0.3 (0.002–2.9)	0.04 (0–0.91)	0.13	0.4557
AUC SS	9.5 (0.04–82)	1.7 (0.06–35)	0.18	0.3176
AT SS	0 (0–115.5)	0 (0–115.5)	0	>0.9999
C_{max} Liver	0.002 (0–0.05)	0.0004 (0–0.01)	0.2	0.6358
AUC Liver	0.04 (0–2.6)	0.009 (0–0.25)	0.225	0.6358
AT Liver	82.50 (16.5–115.5)	82.50 (33–115.5)	0	0.9038
C_{max} CNS	102 (96–109)	101 (98–106)	0.99	0.4557

AUC CNS	11484 (9992–12200)	11526 (11106–11922)	1.00	0.8048
Cranial-to-Caudal ratio	1.2 (0.18–4.3)	1.6 (0.38–4.5)	1.33	0.4557
Data are presented as median (min-max). Ratios between the treatment groups are presented for AUC and C_{max}, while differences between medians are presented for AT. Statistical comparisons were carried out using two-tailed Mann–Whitney test. AT, arrival time (minutes); C_{max}, maximum activity; AUC, area under the time-activity curve; ICS, intracranial space; CS, cervical spine; TS, thoracic spine; LS, lumbar spine; SS, sacral spine. ISO, isoflurane 1.5-2%. K/DEX, ketamine, 100 mg/kg; dexmedetomidine.				

References

1. Blomqvist KJ, Skogster MOB, Kurkela MJ, Rosenholm MP, Ahlstrom FHG, Airavaara MT, Backman JT, Rauhala PV, Kalso EA, Lilius TO: Systemic hypertonic saline enhances glymphatic spinal cord delivery of lumbar intrathecal morphine. J Control Release 2022; 344: 214-224
2. Lee H, Xie L, Yu M, Kang H, Feng T, Deane R, Logan J, Nedergaard M, Benveniste H: The Effect of Body Posture on Brain Glymphatic Transport. J Neurosci 2015; 35: 11034-44
3. Persson NDÅ, Lohela TJ, Mortensen KN, Rosenholm M, Li Q, Weikop P, Nedergaard M, Lilius TO: Anesthesia Blunts Carbon Dioxide Effects on Glymphatic Cerebrospinal Fluid Dynamics in Mechanically Ventilated Rats. Anesthesiology 2024; 141: 338-352
4. Sigurdsson B, Hauglund NL, Lilius TO, Mogensen FL, Mortensen KN, Beschorner N, Klinger L, Baerentzen SL, Rosenholm MP, Shalgunov V, Herth M, Mori Y, Nedergaard M: A SPECT-based method for dynamic imaging of the glymphatic system in rats. J Cereb Blood Flow Metab 2023; 43: 1153-1165
5. Lilius TO, Mortensen KN, Deville C, Lohela TJ, Stæger FF, Sigurdsson B, Fiordaliso EM, Rosenholm M, Kamphuis C, Beekman FJ, Jensen AI, Nedergaard M: Glymphatic-assisted perivascular brain delivery of intrathecal small gold nanoparticles. Journal of Controlled Release 2023; 355: 135-148
6. Lilius TO, Rosenholm M, Klinger L, Mortensen KN, Sigurdsson B, Mogensen FL, Hauglund NL, Nielsen MSN, Rantamaki T, Nedergaard M: SPECT/CT imaging reveals CNS-wide modulation of glymphatic cerebrospinal fluid flow by systemic hypertonic saline. iScience 2022; 25: 105250
7. Yushkevich PA, Piven J, Hazlett HC, Smith RG, Ho S, Gee JC, Gerig G: User-guided 3D active contour segmentation of anatomical structures: significantly improved efficiency and reliability. Neuroimage 2006; 31: 1116-28
8. Tustison NJ, Avants BB, Cook PA, Zheng Y, Egan A, Yushkevich PA, Gee JC: N4ITK: improved N3 bias correction. IEEE Trans Med Imaging 2010; 29: 1310-20
